# Fear Not While Acting Justly

**Published:** 1874-05

**Authors:** 


*" Fear Not while Acting Justly."*

Dr. C.: We sympathise with you in your recent troubles, but be not discouraged—life must have its trials. After all a good man may do in the cause of truth and the right, even then an unjust punishment may be inflicted upon him. It is pleasant to know, however, that such troubles that beset the good man prove at last to be the chained lions of good old Bunyan. God is not asleep; He loves his children; He is on their side against all enemies, whether in the shape of flesh or devils; and, although he may not slay them, as He did the two hundred and fifty princes who rebelled against Moses and Aaron, "with fire from the Lord," or "open the earth and swallow them," as he did Korah Dathan, and Abiram, still God lives and reigns; and he has declared that though hand join in hand, the wicked shall not go unpunished. We are not surprised to hear of certain difficulties. Those who endorse fraud and corruption in others are apt to commit them, and and prove alike unworthy the confidence of honest men. P.

